# Family caregiving experiences of medical school faculty: high prevalence, high strain, and low resource awareness

**DOI:** 10.1186/s12960-024-00944-7

**Published:** 2024-11-12

**Authors:** Kimberly A. Skarupski, David L. Roth, Samuel C. Durso

**Affiliations:** Department of Medicine, Division of Geriatric Medicine and Gerontology, Johns Hopkins University, 2024 E. Monument St., Suite 2-1000, Baltimore, MD 21287 USA

**Keywords:** Academic medicine, Caregiving, Workforce, Resources, Employer-benefits

## Abstract

**Background:**

Adult caregiving can be demanding and stressful, especially when the caregiver is employed. As the age of the U.S. population and workforce increases, more adults are providing care to aging family members.

**Objective:**

To understand the prevalence and aspects of the caregiving experience and caregiving strain among department of medicine faculty members, and to gauge their awareness and utilization of caregiving resources.

**Design:**

We used a cross-sectional survey design. A questionnaire survey was developed and launched in Redcap in October, 2022, and an invitation was emailed followed by two reminders to all full-time and part-time faculty members (*N* = 1053) in our department of medicine.

**Main measures:**

Faculty demographics, caregiver status, caregiving details, degree of mental or emotional strain, and knowledge of and use of employer and external caregiver resources.

**Key results:**

Of the 1053 faculty members who received up to three email survey invitations, 209 (20%) responded of which 76 (36%) were current caregivers and 117 (56%) were non-caregivers. Among the 76 current caregivers, 53 (70%) reported providing care for parents or parent-in-laws and 9 (12%) reported caring for a spouse. One-third of current caregivers reported caring for individuals with Alzheimer’s disease or dementia/memory problems. Ninety-five% of current caregivers reported some or a lot of caregiving strain. A wide variation in knowledge of and use of employer and external caregiver resources was reported.

**Conclusions:**

Department of medicine faculty who provide adult caregiving report a high prevalence of strain and wide variation in knowledge of and use of employer and external caregiver support services, suggesting opportunity to better understand where gaps exist in providing support for caregivers.

## Introduction

The average age of full-time medical school faculty members (*N* = 197,327) in the U.S. is 49.2 years [1] and faculty members age 50 or older comprise 43.3% of the full-time medical school faculty population [2]. Increased age is associated with increased adult caregiving responsibilities. According to a 2017 U.S. Senate special committee on aging report [3], one out of every four employees over the age of 50 serves as a family caregiver and a 2023 Bureau of Labor Statistics report [4] showed that 61.4% of caregivers during 2021–2022 were employed either full or part-time. Caregiving is often demanding and can be stressful, especially when the caregiver is employed.

We tend to associate the term ‘childcare’ when we think about caring for young children and the term ‘caregiving’ when we think about caring for older adults. Both caregiving experiences have similarities and some significant differences. Childcare responsibilities and activities typically: (a) result after some amount of planning and preparation, minimally during the nine months’ gestation period; (b) occur in a co-residing household with the child/children; and (c) include any number of community or employer-provided childcare options (e.g., nannies, au pairs, day care, after-care programs, babysitters, etc.). However, adult caregiving responsibilities and activities may: (a) result after an unplanned medical emergency, accident, or other significant life—or death—event; (b) occur remotely (cross-nationally or internationally) and require frequent travel and/or relocations; and (c) be thwarted by lack of resource awareness in the care recipients’ community and/or inadequate or inaccessible resources. Given these unique challenges that accompany adult caregiving, the experience is oftentimes associated with significant strain and stress.

In our 2021 paper [5], we reported results from a secondary data analysis of a survey of 2126 full-time medical school faculty age 55 and older (average age 62.3) from 14 U.S. LCME-accredited medical schools, conducted in 2017. We found that 19% of the respondents reported providing care on an on-going basis to a family member, friend, or neighbor with a chronic illness or disability, including 22.4% of the female respondents and 17.3% of the male respondents. Among those who reported caregiving, 90.2% reported experiencing some or a lot of mental or emotional strain from caregiving.

In this paper, we report the results from a more extensive survey of caregiving experiences among the faculty members in our department of medicine. Our survey objectives were to understand the caregiving experiences of our institution’s faculty members, including prevalence and aspects of caregiving and caregiver strain, and to gauge their awareness and utilization of caregiving resources.

## Methods

This project utilized a cross-sectional survey design. We developed and launched a questionnaire survey in Redcap in October, 2022. We emailed an invitation and two reminders to all full-time and part-time faculty members (*N* = 1053) in our department of medicine. We excluded faculty with only adjunct, emeritus, or secondary appointments (*n* = 412) to avoid potential confounding related to both employment status and access to employer-based caregiving benefits and resources.

In the emailed survey invitation, we described the survey as follows: “The following confidential survey is an important opportunity for you to share your experiences with disability in your family and if that has affected your work as a faculty member in the Johns Hopkins Department of Medicine. The goal of the survey is to better understand the experiences of Johns Hopkins faculty members and their awareness of resources to help families dealing with disabilities. Whether you are providing care for someone with a disability now, or have done so in the past, or have disabilities of your own, or have never had any of these experiences, we would appreciate your participation in our brief survey. It should take you between 5 and 20 min to complete the survey, depending on your experiences and responses. Your participation in this on-line survey is purely voluntary, and you may discontinue at any time. The survey responses will be analyzed by statisticians in the Johns Hopkins Center on Aging and Health, and your responses to all questions will be kept completely confidential and will not be disclosed to any of your colleagues or supervisors.” The study was exempted by our institutional review board and categorized as a quality improvement project.

In the questionnaire, we asked three primary questions: (1) “Are you currently providing care on an on-going basis to any family member, friend, or neighbor with a chronic illness or disability? This would include any kind of regular help with basic activities such as dressing, bathing, grooming this person, preparing meals, managing bills, arranging for or helping to coordinate medical care, managing medications, watching or supervising this person, or providing transportation.” We also asked, “Do you have any family members or friends with chronic health conditions or disabilities that you would like to provide help to but don’t have enough time to do so?; (2) “How much of a mental or emotional strain is it on you to provide this care?”; and (3) “Are you aware of this (*listed*) resource, have you used it in the past, do you use it currently, or do you anticipate using it, and rate your satisfaction.”

In the resource lists, we identified caregiving resources available to Hopkins employees, namely: (a) Backup Care: reduced fees for in-home care through Care@Work; (b) MySupport: up to 5 counseling sessions; (c) Lifemart: employees discounts for senior care products; (d) dependent care flexible spending accounts; and (e) Hopkins-assisted referrals to external agencies such as Area Agencies on Aging and Eldercare Locator). We also included a list of external/community-based caregiving resources, namely: (a) respite care such as adult day services; (b) home health care/visiting nurses’; (c) caregiver-focused counseling services or support groups; (d) meal services such as Meals on Wheels; (e) transportation services specifically for persons with disabilities; and (f) other domestic services for persons with disabilities, such as shopping, cooking, housework, and yardwork.

For faculty members who reported caregiving responsibilities, we also inquired about caregiving details, such as: relationship of caregiving recipient (e.g., spouse, mother, father, etc.); number of caregiving recipients; whether the faculty member was/is the primary caregiver; if the caregiving recipient lived/lives with the respondent; age of care recipient; if the care recipient has Alzheimer’s disease or dementia/memory problems and other major health conditions/disabilities; and if the care recipient needed help with activities of daily living (e.g., dressing, bathing, toileting, etc.) or instrumental activities of daily living (e.g., money, telephone, meal preparation, etc.); and the number of hours per week spent caregiving. The caregiving questions were adapted from validated instruments used in previous research [6].

Finally, we included standard sociodemographic questions such as age, gender, race, ethnicity, marital status, degree type, full-time or part-time status, number of years at Hopkins, and rank. We tabulated univariate statistics for the total sample (*N* = 1053) using SAS Version 9.4.

## Results

Of the 1053 faculty members who received up to three email survey invitations, 830 (79%) did not respond and of the 223 (21%) respondents, 14 opted-out, which resulted in 209 total survey respondents (20% response rate). Table 1 shows the sociodemographic characteristics of the sample. Of the 209 respondents, 76 (36%) indicated that they were current caregivers and 117 (56%) were non-caregivers (16 [8%] indicated that they had been a caregiver within the prior year; these data were excluded from subsequent analyses). On average, faculty caregivers had been at Hopkins for 18 years, were 54 years of age, were female (63%), non-Hispanic (92%), white (63%), married (87%), with MD degrees (82%), working full-time (78%), and assistant professors (30%), associate professors (24%), and professors (32%). This caregiver profile roughly approximates the total departmental survey sample; that is, relative to the total department, survey respondent caregivers have more years at Hopkins, are slightly older, and disproportionately more female and at part-time status (not tested statistically).

**Table 1:** Faculty survey respondent sociodemographics, by caregiving type

	Total (*N* = 209)	Current CGs (*n* = 76 [36%])	Non-CGs^a^ (*n* = 117 [56%])	
			Wishful CGs (*n* = 30 [14% of total])	Not caregiving (no one needs their help) (*n* = 87 [42% of total])
Years at Hopkins	15.8 (11.3)	17.9 (10.9)	15.2 (9.0)	14.4 (12.5)
Age				
Mean (SD)	51.1 (12.0)	54.0 (11.0)	48.6 (8.9)	49.7 (13.7)
Range	30–81	34–79	35–65	30–81
Female gender, *number (%)*	112 (54.9)	47 (62.7)	18 (64.3)	38 (44.2)
Male	97 (45.1)	29 (37.3)	12 (35.7)	49 (55.8)
Race				
African American	9 (4.3)	4 (5.3)	1 (3.3)	3 (3.5)
Asian/Pacific Islander	38 (18.2)	15 (19.7)	9 (30.0)	13 (14.9)
Mixed/Other/Missing	18 (8.6)	9 (11.8)	1 (3.3)	6 (6.9)
White	144 (69.0)	48 (63.2)	19 (63.3)	65 (74.7)
Non-Hispanic	199 (95.2)	70 (92.1)	30 (100.0)	84 (96.6)
Marital status				
Married	177 (85.9)	64 (86.5)	27 (90.0)	72 (83.7)
Cohabitating	8 (3.9)	3 (4.1)	1 (3.3)	4 (4.7)
Widowed	3 (1.5)	1 (1.4)	–	1 (1.2)
Divorced	8 (3.9)	2 (2.7)	2 (6.7)	3 (3.5)
Separated	3 (1.5)	1 (1.4)	–	2 (2.3)
Single, never married	7 (3.4)	3 (4.1)	–	4 (4.7)
Highest degree completed				
MD	167 (80.7)	62 (81.6)	21 (70.0)	73 (83.4)
PhD or equivalent	27 (13.0)	8 (10.5)	6 (20.0)	11 (12.8)
Masters or equivalent	13 (6.3)	6 (7.9)	3 (10.0)	2 (2.3)
Status				
Full time	175 (83.7)	59 (77.6)	28 (93.3)	74 (85.1)
Part time	34 (16.3)	17 (22.4)	2 (6.7)	13 (14.9)
Rank				
Instructor	30 (14.4)	10 (13.2)	5 (16.7)	13 (14.9)
Research Associate	10 (4.8)	1 (1.3)	4 (13.3)	5 (5.8)
Assistant Prof	70 (33.5)	23 (30.3)	6 (20.0)	34 (39.1)
Associate Prof	43 (20.6)	18 (23.7)	7 (23.3)	15 (17.2)
Professor	54 (25.8)	24 (31.6)	8 (26.7)	19 (21.8)
Professor (PAR)	1 (0.5)	–	–	1 (1.2)
Associate Prof (PAR)	1 (0.5)	–	–	–

PAR: pending appointment at rank

^a^16 respondents (8%) indicated that they had been a caregiver in the prior year (data were excluded)

Among the 117 non-caregivers, 30 (26%) reported that they did have “family members or friends with chronic health conditions or disabilities” whom they did want to provide help for but did not have enough time to do so (i.e., ‘wishful caregivers’) and 87 (74%) reported that they had no family or friends with disabilities needing their help.

Table 2 reports caregiving details by current caregivers. Among the 76 current caregivers, 53 (70%) reported providing care for parents or parents-in-law and 9 (12%) reported providing care for a spouse. When the 76 current caregivers were asked, “how much mental or emotional strain is it on you to provide this care?”, 4 (5%) reported no strain, 46 (61%) reported some strain, and 26 (37%) reported a lot of strain.

**Table 2:** Current caregiving details

	Current CGs (*n* = 76)
Relationship, *number (%)*	
Spouse	9 (12.0)
Mother	30 (40.0)
Father	14 (18.7)
Mother-in-law	6 (8.0)
Father-in-law	3 (4.0)
Son	5 (6.7)
Daughter	1 (1.3)
Sister	2 (2.7)
Brother	2 (2.7)
Grandmother	2 (2.7)
Other	1 (1.3)
How much of a mental or emotional strain is it on you to provide this care? *number (%)*	
No strain	4 (5.3)
Some strain	46 (60.5)
A lot of strain	26 (34.2)
Yes, I am/was caregiving for more than one person, *number (%)*	30 (40.0)
CG for how many people?	
* Mean (SD)*	2.1 (0.5)
* Range*	1–3
Yes, I am the primary CG	41 (54.7)
CG recipient currently lives with me	22 (30.1)
Age of care recipient	
* Mean (SD)*	75.7 (19.5)
* Range*	9–104
Recipient has AD or dementia/memory problems, *yes*	29 (38.2)
Recipient has other major health conditions/disabilities?	
Stroke-related impairment/disabilities *number (%)*	4 (5.3)
Other weakness/physical disability	36 (47.4)
Mental/behav impairments	14 (18.4)
Major sensory impairments	13 (17.1)
Cancer/impairments related to cancer	12 (15.8)
Other	6 (7.9)
Recipient needs help with ADLs? (dressing, bathing, toileting, grooming, eating, in and out of bed)	30 (40.0)
Recipient needs help with IADLs? (money, telephone, meal prep, meds, shopping, transport)	68 (91.9)
Hours per week caregiving*	
* Mean (SD)*	8.8 (18.8)
*Men:11.0 (29.7) hrs vs. Women:7.7 (8.4)—ns range	1–152
CG length of time?	
Years,* mean (SD)*	5.4 (6.7)
Months*, mean (SD)*	2.6 (3.1)

Thirty (40%) of the current caregivers reported that they were caring for more than one person (averages = 2.1). More than half of current caregivers (55%) reported that they were the primary caregiver and 30% of current caregivers reported that their care recipient lives with them. The average age of the current caregivers’ care recipient was 76 (standard deviation = 19.5).

More than one-third (38%) of current caregivers reported that their care recipient had AD or dementia/memory problems. The other major health conditions/disabilities for caregivers were: weakness/physical disability; mental/behavior impairments; major sensory impairments; and impairments related to cancer. Forty percent of current caregivers reported that their care recipient needed help with ADLs and 92% reported that their recipient needed help with IADLs. Current caregivers reported an average of 9 h (sd = 19) per week of caregiving for an average of 5.5 years (sd = 6.7).

Table 3 reports the awareness and utilization of employer-provided or endorsed caregiving-related resources. Current caregivers’ (*n* = 76) awareness of the resource ranged from a low of 8% (“Lifemart: employee discounts for senior care products”) to a high of 69%% (“Dependent care flexible spending account”). Utilization of these employer-sponsored resources ranged from a low of 17% for backup care to a high of 44% for the dependent care flexible spending account. Satisfaction ratings ranged from a low of 2.0 (“Lifemart”) to 3.9 (“Dependent care flexible spending account”) on a 1–5 scale, where 1 is very dissatisfied and 5 is very satisfied.

**Table 3:** Hopkins resources, by current caregivers combined (*n* = 76)

	Yes, I am aware of this resource	Yes, I have used this resource	Yes, I am currently using this resource	Do you anticipate using this resource in the near future?	If you have used this resource, please rate your satisfaction on a 1–5 scale where 1 is very dissatisfied and 5 is very satisfied
				*Yes*	
				*No*	
				*Not sure*	
Backup Care: reduced fees for in-home care through Care@Work	23 (30.7)	4 (17.4)	1 (25.0)	2 (8.7)	3.8 (0.5) *n* = 4
				10 (43.5)	
				11 (47.8)	
MySupport: up to 5 in-person counseling sessions via telephone	22 (30.1)	8 (36.4)	1 (12.5)	1 (4.6)	3.1 (1.1) *n* = 8
				11 (50.0)	
				10 (45.5)	
Lifemart: employee discounts for senior care products	6 (8.1)	2 (33.3)	–	–	2.0 (–) *n* = 1
				2 (33.3)	
				4 (66.7)	
Dependent care flexible spending accounts	50 (69.4)	22 (44.0)	7 (31.8)	10 (20.0)	3.9 (1.1) *n* = 20
				27 (54.0)	
				–	
Hopkins-assisted referrals to external agencies such as Area Agencies on Aging and Eldercare Locator	11 (15.1)	4 (36.4)	1 (25.0)	2 (18.2)	3.8 (1.9) *n* = 4
				5 (45.5)	
				4 (36.4)	

Other (specify): Freq = 1 each for: Care.com; case manager at geriatrics clinic; hired in-home help; JHHC/Capable (research study) / physicians; local care staffing agencies; Alz Assn.; place for mom and Aetna

When presented with the list of external caregiving resources (Table 4), nearly half (45%) of the current caregivers indicated that they had utilized home health care/visiting nurses, more than one-quarter (26%) reported having utilized other domestic services for persons with disabilities (e.g., shopping, cooking, housework, yardwork), and the satisfaction scores ranged from 3.7 for other domestic services to 5.0 for respite care such as adult day services.

**Table 4:** External resources, by current caregivers (*n* = 76)

	Yes, I have used this resource	Yes, I am currently using this resource	Do you anticipate using this resource in the near future?	If you have used this resource, please rate your satisfaction on a 1–5 scale where 1 is very dissatisfied and 5 is very satisfied
			*Yes*	
			*No*	
			*Not sure*	
Respite Care such as adult day services	1 (1.4)	1 (100.)	6 (8.2)	5.0 (−) *n* = 1
			39 (53.4)	
			28 (38.4)	
Home health care/visiting nurses	33 (44.6)	12 (36.4)	24 (32.9)	3.8 (1.0) *n* = 29
			21 (28.8)	
			28 (38.4)	
Caregiver-focused counseling services or support groups	1 (1.4)	–	7 (9.6)	4.0 (–) *n* = 1
			36 (49.3)	
			30 (41.1)	
Meal services such as Meals on Wheels	6 (8.1)	3 (50.0)	3 (4.1)	4.3 (0.5) *n* = 6
			54 (74.0)	
			16 (21.9)	
Transportation services specifically for persons with disabilities	6 (8.1)	4 (66.7)	8 (11.0)	4.0 (1.0) *n* = 5
			42 (57.5)	
			23 (31.5)	
Other domestic services (e.g., shopping, cooking, housework, yardwork) for persons with disabilities	19 (25.7)	15 (79.0)	22 (29.7)	3.7 (1.0) *n* = 18
			26 (35.1)	
			26 (35.1)	

Other (specify): Freq = 1 each for: 24-h in-home care; memory care unit in Boston; personal care homes (supervised residential living) and nursing homes (SNFs); health aide; church community; hospice; infusion pharmacy; private duty nurse; self-paid caregivers

## Discussion

Through this work, we learned about caregiving experiences among faculty members in a school of medicine. In brief, we report three major findings: (1) a high rate of caregiving; (2) high caregiving strain among caregivers; (3) general low awareness and utilization of employer-provided/sponsored resources and somewhat higher utilization of external, community-based resources. In our department of medicine, we found that more than one-third of the full-time and part-time faculty survey respondents reported that they were currently providing care on an on-going basis to a family member, friend, or neighbor with a chronic illness or disability (9 h a week, on average) and more than half indicated that providing this care resulted in ‘some mental or emotional strain’ while more than one-third reported ‘a lot of strain’.

Globally, longevity is increasing. The World Health Organization (2022) estimates that by 2030, one in six people in the world will be age 60 or older and by 2050, the population of those age 60 or older will double to 2.1 billion [7]. As we age, we inevitably face health challenges that may require some degree of caregiving, such as episodic events requiring short-term hospital or rehabilitation stays or chronic diseases or conditions requiring long-term care, assisted-living, home nurse visits, and/or specialized care treatments for Alzheimer’s/dementia, cancers, renal failure, and other serious chronic conditions. Spouses, partners, and family members are integrally involved in these caregiving life events. In fact, family and unpaid caregivers provide most of the actual care for older adults [8, 9]. Furthermore, many of these family caregivers are employed full-time [10]. Additionally, our population’s lower fertility rates have reduced the number of available siblings or family members to assist with caregiving [11] and because academic medicine faculty members are typically a geographically mobile population, their aging parents and relatives may live across the country or around the world, making caregiving an even greater challenge and time-commitment. Indeed, we found a small group of faculty members we called “wishful caregivers” who would have liked to provide help to a family member or friend with a chronic condition or disability, but did not have the time to do so. Furthermore, we also observed that 16 (8%) of the survey respondents reported that within the past year they *had* been a caregiver (data not reported here) which uncovers a segment of faculty/employees whose caregiving experiences may have altered/may alter their work roles and expectations. Finally, although women have historically borne the brunt of caregiving, recent data indicate that more men are taking on caregiving responsibilities. In our prior work [5], we showed that 22% of the women faculty reported being caregivers and 17% of the men also reported being caregivers. In their national analysis of family caregivers of older adults from the period of 1999–2015, Wolff et al. [12] also observed an increasing trend of males being caregivers (from 32% in 1999 to 36% in 2015). That said, the economic impact of caregiving is substantial and particularly acute for women. In 2023, the Urban Institute [13] estimated that women who engage in adult or child caregiving forgo nearly $300,000 in lifetime earnings due to both reduced income and retirement earnings and Skira [14] estimated that the welfare cost of caring for an older parent is approximately $165,000 over two years. Caregiving obligations may result in fewer full-time or tenured faculty who are women which may impact the next generation of physicians.

In addition to the general demographic trends, it has been estimated that by 2034, there will be a shortage of between 37,800 and 124,000 physicians [15]; the primary driver is population growth and aging. This underscores a likely future of increased competition by schools of medicine to recruit and retain their faculty workforce. Increasing age of medical school faculty and the population—in addition to other demographic trends—will inevitably result in increased caregiving demand on faculty time, finances and energy. Ensuring that faculty have the resources to provide for a predictable increase in caregiver demands should become one component of a strategy to maintain well-being and optimize work performance.

Our institutional human resource offices post resources on websites for employees. Unfortunately, many faculty member caregivers report that the website is difficult to find and navigate. Furthermore, when they do find the websites and traverse myriad links, faculty members commonly complain about their experiences. For example, with childcare, faculty members report struggling to identify the appropriate resource(s) and then having trouble securing safe, reliable, and affordable childcare. The experiences of faculty members who are seeking the same safe, reliable, and affordable care for their older loved ones, who oftentimes live geographically distant, can be even more frustrating. Resources to support faculty members who have to and who want to provide care for their parents, parents-in-law, other older family members, spouses, partners should both meet their needs and be non-burdensome to access. If employees had accessible and reliable care for their loved ones, they may choose to remain in the workforce longer and/or maintain full-time work status for a longer period of time, which would benefit the institutions. Some state Medicaid plans offer resources that universities could model, such as: self-directed personal assistance services, structured family caregiving (e.g., adult foster care, adult family living, monitored in-home caregiving, coordinated caregiving), and the caretaker child exception (allows an adult child to be ‘paid’ for providing care assistance for an aging parent) [16]. The anxiety and frustration that caregivers experience could be alleviated by centralized and facilitated access to vetted caregiving services and appropriate benefits. Normalizing the utilization of resources may also mitigate the perceived stigma around work flexibility and likely improve employee morale, engagement, recruitment, and retention.

In this survey, we did observe that the lowest prevalence of full-time employment (78%) was among current caregivers. Put another way, 22% of the current caregivers reported part-time employment, compared to 16% of the total departmental survey sample, 7% of the ‘wishful caregivers,’ and 15% of the non-caregivers. This may suggest that caregiving employees may be choosing to or are forced to reduce their employment effort because they *have to* or *want to* provide care for their loved one(s). However, it may also be true that part-time clinical or teaching faculty may not have completed the survey instrument at the same rate as full-time or research-focused faculty members, further *under*-estimating the number of part-time faculty caregivers.

Alternatively, faculty members who are not working full-time may also be more likely to take on caregiving responsibilities.

Interestingly, almost one-third of the caregivers were at the rank of professor, higher than in the overall sample (26%), the ‘wishful caregivers’ (27%), and the non-caregivers (22%). It is unclear whether the demands of caregiving preceded, occurred concurrently, or occurred after being promoted to professor and/or full-time or part-time employment status. Future work in this area would be improved by interrogating the intersections of caregiving demands and institutional expectations of faculty members. A recent comprehensive National Academy of Sciences, Engineering, and Medicine report entitled: “Supporting Family Caregivers in STEMM: A call to action (2024)” [17] highlights how organizational ‘cultural schemas’ and outdated assumptions ensure that stereotypes of the academic workaholic persist. The report describes how academia’s policies and expectations around promotion timelines, early-career grant funding, and participating in professional conferences and societies may collide with child-bearing, child-care leave, and childcare (we would furthermore note that expectations for later-career productivity, mentorship, and leadership collide with their caregiving responsibilities for older adults). In addition, the authors note that other informal cultural schemas such as scheduling meetings and presentations early in the morning or later in the day further support the notion that faculty have no (or should have no) competing family obligations and interests.

### Call for resources, policies, and programs

We urge medical schools to assess the caregiving needs of their faculty and identify and vet caregiving resources and support. We offer four suggestions related to resources, policies, and programs.

First, to improve access to resources, institutions and/or schools of medicine might invest in new *human resource concierge-type positions* to help faculty navigate the myriad caregiving policies, programs, and resources. For example, many of our institutions’ libraries have ‘informationists’ (there are 13 informationists at the Johns Hopkins University Welch Medical Library [18]). Informationists provide information to faculty, staff, and students via their unique skills, expertise, and access to databases and materials not available to the general public. Informationists work with faculty members to clarify the: research question(s) and hypotheses; relevant population/sample; clinical/scientific field and context; span of years; identify other possible related search terms; etc. This service: (a) minimizes barriers to research and scholarship by saving faculty members’ precious time; (b) contributes to operational and financial efficiencies by appropriately stewarding higher-paid faculty salaries to tasks other than literature searches (i.e., clinical work, other research, education, administrative, and service responsibilities); (c) increases faculty confidence in having acquired all the current and relevant literature; and (d) increases morale and faculty satisfaction. A caregiving concierge with similar (caregiving) skills, expertise, and access to resources would accomplish the same outcomes for faculty members around national and international home health care providers, adult day services, and care coordination options: (a) time saved; (b) improved efficiencies; (c) increased confidence in knowing all the resources available to them and their loved ones; and (d) increased morale and faculty satisfaction, likely resulting in improved retention.

Second, institutions and/or schools of medicine could reexamine their child-care and elder-care policies for adequate: paid leaves of absence; mini-sabbaticals; temporary part-time employment arrangements; flexible schedules; and tenure-clock freezes. A network of universities and other academic institutions would be well-suited to pilot these or other novel programs and share from this learned experience. The Family and Medical Leave Act (FMLA) [19] entitles eligible employees to take up to 26 weeks of *unpaid*, job-protected leave for specified family and medical reasons.

Third, in addition to resources and policy changes, institutions could provide and/or facilitate regular programming for faculty member caregivers. Our school of medicine hosts a monthly ‘virtual caregiving community’ (attendance ranges from 6 to 10 faculty caregivers per month) with the goals of: (a) sharing caregiving experiences, critical issues, and lessons learned; (b) curating a host of resources around home health care providers, adult day services, and care coordination; and (c) supporting and encouraging each other. Other faculty career development programs include: retirement panel presentations; a three-part ‘Next Chapter” series exploring life after full-time employment; and one-on-one coaching. These and other programs work to normalize the caregiving experience in the work culture.

Fourth, institutions should systematically collect and monitor data related to: resource utilization and experiences; caregiving status; caregiving strain; anticipated caregiving status for others and for self; intention to retire/exit; and factors associated with retirement/exit. These data could be systematically collected as part of regular faculty satisfaction surveys, faculty well-being, and/or other survey cycles. When data are not measured, false assumptions about faculty members’ wants and needs are more likely. For example, in recent publications, we found that faculty members’ and dean-level leaders’ perceptions about expectations to retire and factors associated with retirement are disparate [20, 21]. More systematic data collection and transparent data sharing about such factors are likely to better inform institutional policies and support employee morale. The NASEM report [17] complements the recommendations we make here and amplifies the facts that caregiving is oftentimes: invisible, undervalued, stigmatized, and a barrier to career success. The report also concurs that best practices in policy implementation and design are not well-communicated and people are unaware of their university’s resources. Similarly, the report calls for innovation in caregiving support—hence our suggestion of human resource concierge-type positions.

## Limitations

This study is limited by our access to key faculty characteristics at the department and school levels. For example, we did not query sociodemographic data for the department and school at the same time we conducted our survey, limiting our ability to make inferences about the faculty caregiving experience more broadly. Nonetheless, relative to the entire department of medicine (*N* = 1053) and the school of medicine (*N* = 5130), our survey sample was the same average age (51), and had similar proportions at the assistant, associate, and professor ranks (within 5 percentage points [data not reported here]). However, our survey sample had more full-time (84%) and women (55%) respondents relative to the school (66% and 47%, respectively [data not reported here]). We were also unable to ascertain the timing of caregiving demands relative to promotions and/or changes in employment status.

In our prior work [5], we reported that 19% of the nationally representative faculty respondents reported being a caregiver; however, that caregiving question was embedded in a national survey instrument designed to measure expectations about retirement. This current survey instrument was focused solely on caregiving, likely garnering the attention of faculty members for whom the topic was especially relevant, perhaps making the 36% caregiving rate an over-estimate. However, it is also likely that at least some of these 830 non-respondents were also caregivers, rendering the 7.2% rate an underestimate. We have no way of knowing the true rate of caregiving among faculty members; we surmise that 15–20% is a realistic estimate based on others’ and our prior work [5, 22].

## Conclusion

Caregiving demands for medical school faculty mirror those of the general population and stress associated with those demands impacts work productivity and satisfaction. Competitive pressure to recruit and retain the best medical school faculty will require understanding the needs of faculty providing or wishing to provide caregiving and then providing adequate and accessible resources that meets those needs.

## Data Availability

The data that support the findings of this study are available from the authors but restrictions apply.

## Abbreviations

LCME: Liaison Committee on Medical Education
FMLA: Family and Medical Leave Act
